# Identification of a Peptide Produced by *Bifidobacterium longum* CECT 7210 with Antirotaviral Activity

**DOI:** 10.3389/fmicb.2016.00655

**Published:** 2016-05-04

**Authors:** Empar Chenoll, Beatriz Casinos, Esther Bataller, Javier Buesa, Daniel Ramón, Salvador Genovés, Joan Fábrega, Montserrat Rivero Urgell, José A. Moreno Muñoz

**Affiliations:** ^1^Department of AgroFood Biotechnology, Biópolis S.L.Valencia, Spain; ^2^Department of Microbiology, School of Medicine, University of Valencia – Hospital Clínico UniversitarioValencia, Spain; ^3^Laboratorios Ordesa S.L.Barcelona, Spain

**Keywords:** *B. longum* subsp. *infantis* CECT 7210, probiotics, rotavirus, 11-mer peptide, protease

## Abstract

Rotavirus is one of the main causes of acute diarrhea and enteritis in infants. Currently, studies are underway to assess the use of probiotics to improve rotavirus vaccine protection. A previous work demonstrated that the probiotic strain *Bifidobacterium longum* subsp. *infantis* CECT 7210 is able to hinder rotavirus replication both *in vitro* and *in vivo*. The present study takes a systematic approach in order to identify the molecule directly involved in rotavirus inhibition. Supernatant protease digestions revealed both the proteinaceous nature of the active substance and the fact that the molecule responsible for inhibiting rotavirus replication is released to the supernatant. Following purification by cationic exchange chromatography, active fractions were obtained and the functional compound was identified as an 11-amino acid peptide (MHQPHQPLPPT, named 11-mer peptide) with a molecular mass of 1.282 KDa. The functionality of 11-mer was verified using the synthesized peptide in Wa, Ito, and VA70 rotavirus infections of both HT-29 and MA-104 cell lines. Finally, protease activity was detected in *B. longum* subsp. *infantis* CECT 7210 supernatant, which releases 11-mer peptide. A preliminary identification of the protease is also included in the study.

## Introduction

Group A rotavirus is one of the major causes of acute gastroenteritis in infants and young children, leading to an estimated 2 million hospitalizations per year (Parashar et al., 2006). Despite the administration of rotavirus vaccines, rotavirus still accounts for an estimated 21.3–39.5% of the hospital admissions for diarrheal disease in Europe (Williams et al., 2009). However, hospitalizations of older children and adults for diarrhea have greatly decreased after the introduction of infant rotavirus vaccination, which suggests that some rotavirus episodes were transmitted from infants (Lopman et al., 2011). Rotaviruses are transmitted by the fecal-oral route, inducing diarrhea due to malabsorption by the infected epithelial cells of the intestinal mucosa (Kapikian et al., 2001) and increased intestinal motility (Istrate et al., 2014). The mechanisms underlying diarrhea include secondary malabsorption, patchy lesions, villous atrophy, and disruption of the tight junctions in the small intestine, altered ion channel transport due to the secretion of neurotransmitters (Hagbom et al., 2012), NSP4 toxicity (Hodges and Gill, 2010), the activation of the enteric nervous system (Lundgren et al., 2000), and the expression of aquaporins (Cao et al., 2014). The process is characterized by profuse diarrhea, which can cause severe dehydration and, in the case of non-intervention, lead to death (Parashar et al., 2006). Several vaccines have been developed, demonstrating different degrees of protection against rotavirus infection (Vesikari et al., 2007; Dennehy, 2008; Glass et al., 2014). Nevertheless, it has been reported that despite wide coverage, the antibodies induced by vaccination cannot totally prevent symptomatic infections of some rotavirus genotypes (Bucardo et al., 2015). These findings imply that new generation vaccines and complementary preventive approaches are needed to decrease the morbidity and mortality associated with rotavirus diarrhea (Demirjian and Levy, 2009; Glass et al., 2014).

During the last few years, research has focused on the potential use of probiotics as preventive agents against rotavirus. The Food and Agriculture Organization (FAO) and the World Health Organization (WHO) define probiotics as “live microorganisms which when administered in adequate amounts confer a health benefit on the host” (FAO/WHO, 2001). These microorganisms are demonstrated to be effective in preventing several disorders such as infections, diarrhea, and inflammatory diseases (Reid, 2008; Kotzampassi and Giamarellos-Bourboulis, 2012). Moreover, probiotics affect the modulation and establishment of intestinal microbiota and enhance maturation of the innate and adaptive immune systems among others (Kotzampassi and Giamarellos-Bourboulis, 2012). Regarding the efficacy of these microorganisms in treating rotavirus-related diarrhea, a number of probiotics have been reported including *Bifidobacterium longum* and *Lactobacillus acidophilus* strains (Lee et al., 2014) and commercial probiotic strains such as *Lactobacillus* GG (Sindhu et al., 2014). Nevertheless, most of these reports are based on the amelioration of diarrhea symptoms but little has been published on the identification of the mechanisms and molecules underlying rotavirus inhibition. In this respect, current ideas regarding these mechanisms of action include changes in gut ecology, an effect on the gut mucosal barrier, and a modulation of the immune response (Kotzampassi and Giamarellos-Bourboulis, 2012), each being strain-specific (Oelschlaeger, 2010).

A previous study demonstrated that strain *B. longum* subsp. *infantis* CECT 7210 exerts a direct *in vitro* effect on rotavirus infection in HT-29 and MA-104 cell lines (Muñoz et al., 2011). Furthermore, an *in vivo* effect was shown to involve both antiviral effects and immunological enhancement, providing preliminary protection against the rotavirus infection in a mouse model. The strain was also characterized, demonstrating that *B. longum* subsp. *infantis* CECT 7210 fulfills the main criteria required for consideration as a probiotic. However, the interaction of the probiotic with the host cell surface alone did not explain all the mechanisms involved, since infection levels decreased even when the probiotic was added directly to the virus, therefore further studies must be conducted to establish the mechanisms involved in the antiviral effect of this probiotic strain.

Thus, the aim of this study is to identify the molecule(s) directly responsible for the activity of strain *B. longum* subsp. *infantis* CECT 7210 against rotavirus and to investigate the mechanism underlying the inhibition.

## Materials and Methods

### Cells and Viruses

The human colon carcinoma cell line HT-29 was grown in Dulbecco’s modified minimal essential medium (DMEM) supplemented with 10% (v/v) fetal bovine serum (FBS, Gibco Invitrogen, Paisley, UK). The Rhesus monkey kidney cell line MA-104 was grown in Eagle’s minimal essential medium (MEM) supplemented with 10% (v/v) FBS. Human rotavirus strain Wa (G1-P1A[8]) and simian rotavirus strain SA11 (G3-P1A[8]) were obtained from the American Type Culture Collection. Human rotavirus strain VA70 (G4-P1A[8]) and porcine rotavirus strain Ito (G3-P1A[8]) were a gift from Prof. Albert Bosch (University of Barcelona, Spain). *B. longum* subsp. *infantis* CECT 7210 is a probiotic strain isolated from baby’s feces and identified and characterized previously by Muñoz et al. (2011)

### Rotavirus Propagation and *In Vitro* Inhibition Assays

Assays were carried out as in Muñoz et al. (2011). Competition assays were performed with rotavirus SA11, Ito, VA70 and Wa in both HT-29 and MA-104 cell lines. In all cases, cell monolayers were grown in 96-well plates and the tested compounds were incubated with rotavirus followed by the infection of the cells with this virus-compound mixture (strategy A) or the compound was incubated with the cell cultures followed by the inoculation of the virus (strategy B). In both cases, viral antigens were detected by immunoperoxidase assays (Muñoz et al., 2011). Infectious peroxidase-stained foci were counted and arithmetic means were calculated to determine the number of foci per microscopic field, which were compared with the number of infectious foci of untreated virus controls to obtain the reduction percentage of virus focus-forming units. The identified peptide was synthesized at 90% purity (Genscript Corporation, Piscataway, NJ, USA) and its activity was measured for both strategies at different concentrations (1, 5, 10, and 50 μM).

### Purification and Identification of the Substance/s of Interest by Cationic Exchange Chromatography Followed by Reverse-Phase Chromatography

#### Supernatant Processing

A volume of 10 L culture of the strain *B. longum* subsp. *infantis* CECT 7210 growing in MRS (de Man, Rogosa, and Sharpe, CM1153, Oxoid, Basingstoke, UK) medium supplemented with cysteine (0.05% w/v; MRSC) was obtained and centrifuged at 12,000 × *g* for 15 min at 4°C. Supernatant was neutralized to pH 6.5 with NaOH (10N), 10x concentrated by freeze-drying and sterilized by 0.22-μm-pore-size filter (Minisart hydrophilic syringe filter; Sartorius Stedim Biotech GmbH, Gottingen, Germany). Concentrated supernatant was stored at -20°C until use.

#### Identification of the Nature of the Active Compound

In order to study, the nature of the substance of interest, the freeze-dried samples were treated with lipase and proteases (proteinase K and pepsin). To study the effect of lipase, a freeze-dried sample (from 25 mL supernatant) was re-suspended in 2.4 mL phosphate buffer solution (50 mM, pH 6.9), then 0.1 mL lipase from porcine pancreas was added (50 mg/mL; Sigma–Aldrich). The mixture was incubated at 37°C for 2 h, and the reaction was inactivated taking the solution to pH 4.5. Regarding pepsin, a freeze-dried sample (from 25 mL supernatant) was re-suspended in 1.25 mL of McIlvaine buffer 50 mM pH 4.0, to which 0.109 mL of a pepsin solution was added (50 mg/mL; Sigma–Aldrich, St. Louis, MO, USA). This mixture was incubated at 37°C for 2 h, and the reaction was inactivated taking the solution to pH 6.5 in a final volume of 2.5 mL. In the case of proteinase K, freeze-dried sample (from 25 mL supernatant) was re-suspended in 2.4 mL of phosphate buffer 50 mM pH 6.5, to which 0.1 mL of a proteinase K solution was added (50 mg/mL; Sigma–Aldrich). This mixture was incubated at 37°C for 2 h, and then proteinase K was inactivated by incubation at 100°C for 15 min. Analysis of the inhibition of proliferation of rotavirus SA11 was performed in all processed supernatants.

The negative controls included in these tests were supernatant of culture medium MRSC without inoculation (C1) and the same supernatant incubated for 2 h at 37°C (QC3). Control with non-inactivated proteinase K supernatant could not be tested due to post-incubation cell lysis. As a negative control of inhibition, a non-fermented freeze-dried sample was used (C1). As positive control of infection, a solution containing cells and viruses was used.

#### Purification and Identification of the Substance Active against Rotavirus

Purification was performed following Chenoll et al. (2011). Briefly, the supernatant was added to sodium phosphate buffer (20 mM, pH 5.8) and then applied to a cationic exchange column (HiPrep 16/10 SP FF, GE Healthcare, Amersham Biosciences AB, Sweden) by means of a chromatography system (ÄKTA Explorer, Amersham Pharmacia Biotech). All those proteins of cationic nature adsorbed were then eluted with sodium phosphate buffer (20 mM, pH 5.8) and equilibrated with NaCl 1M without gradient. The proteins were eluted simultaneously in a few fractions in minimal volume (10 mL). Each collected fraction was subjected to an ultrafiltration process using 5,000 Da filters (Extreme Amicon, Millipore, Billerica, MA, USA) followed by another stage of reverse phase chromatography with a RESOURCE RPC 3 mL column (GE Healthcare). An aliquot of these fractions was taken to dryness to eliminate the dissolvent and re-suspended in a volume of MEM buffer (Sigma–Aldrich) just before performing *in vitro* inhibition assays. In total, 28 fractions were selected for rotavirus inhibition assays (strategy B) with SA11 virus in MA-104 cell line as explained above. The fractions that gave a positive inhibition result were analyzed by MALDI-TOF peptide mass fingerprinting at the “Centro Nacional de Investigaciones Cardiovasculares” (CNIC, Madrid, Spain), to determine the molecular weight(s) of the peptide(s) present in each fraction. Sequences obtained were compared against the database of proteins/peptides by means of the BLAST online tool.

### Purification and Identification of the Protease

#### Supernatant Processing

A volume of 1 L culture of strain *B. longum* subsp. *infantis* CECT 7210 in MRSC medium was obtained and centrifuged at 12,000 × *g* for 15 min under refrigerated conditions. Proteins were precipitated by the addition of ammonium sulfate (80% saturation) and collected by centrifugation (30 min, 12,000 × *g*). Pellet was resuspended in Tris-HCl buffer (Tris-HCl 20 mM, pH 8.5), dialyzed with a 10 KDa membrane at 4°C and applied to an anionic exchange column (HiPrep 16/10 Q XL, GE Healthcare) by means of a chromatographic system (ÄKTA Explorer). Anionic proteins were eluted with buffer Tris-HCl (20mM, pH 8.5, CaCl_2_ 5 mM) using a gradient of NaCl from 0 to 1 M. As an indicator of the purification process, samples were monitored at 280 nm throughout elution and protease activity measured with BSA as a substrate following Bradford (1976). Protease-positive fractions were then concentrated by 10 KDa ultrafiltration (Millipore), diluted with distilled water and applied to gel filtration chromatography (1% column volume) with a HiLoad Superdex 75 prep grade column (GE Healthcare Europe GmbH, Barcelona, Spain) with fractionation in a range of 3,000–70,000 Da. Sample was monitored with absorbance at 280 nm, and fractions with proteins were collected, desalted by PD10 columns (GE Healthcare Life Sciences) and concentrated by 10 KDa ultrafiltration. A volume of 30 μL of protease-positive fraction was electrophoresed on a 12.5% polyacrylamide SDS-PAGE gel. Gel was stained with silver nitrate and a 209–7.1 KDa commercial molecular weight marker (SDS-PAGE Standards, Broad Range) was used. The single band was separated and analyzed by MALDI-TOF peptide mass fingerprinting at CNIC, to determine the molecular weight and sequence of the protein. Sequence obtained was compared against the database of proteins/peptides by means of the BLAST online tool.

#### Evaluation of 11-mer Production from β-Casein Using the Purified Protease

In order to evaluate whether the purified protease hydrolyzes β-casein protein to obtain 11-mer peptide, an enzymatic activity assay was performed with β-casein as substrate and purified protease from CECT 7210 supernatant as enzyme. The hydrolysis was carried out in a solution containing 15 ml of protease-purified fraction (0.04 U), 30 mL of 2% β-casein solution (BioUltra, Sigma–Aldrich) and 105 mL phosphate citrate buffer (50 mM pH 6.4). After 48 h hydrolysis at 50°C, samples were boiled for 10 min and peptides formation was analyzed by HPLC. Evaluation of 11-mer formation and β-casein degradation was conducted on a HPLC (Waters 2695) with photodiode array detector (Waters 2996) and C18 column (Sunfire Waters C18 5 mm 4.6^∗^150 mm). Eluents were water (MilliQ quality; A) and acetonitrile (90% v/v; B), both with TFA 0.1% (v/v). Chromatographic conditions are summarized in **Table 1**.

**Table 1:** HPLC chromatographic conditions used to quantify peptide 11-mer.

Time (min)	% Buffer A	% Buffer B
–	100	0
40	50	50
42	0	100
50	0	100
60	100	0

### Characterization of the Purified Protease

#### Carbon Sources

Protease activity was assayed in supernatants of strain CECT 7210 grown in MRSC media containing glucose, lactose or maltose as sole carbon source, respectively (all of them at 20 g/L). Protease activity was measured in supernatants after 17 h of growth and centrifuged at 12,000 × *g* for 15 min under refrigerated conditions. BSA degradation (1% w/v, citrate-phosphate buffer 50 mM, pH 6.4) was measured at different incubation times (1 h, 3 h and 5 h) both with the supernatants and with 3 KDa concentrated fractions. Using the supernatants and the 3 KDa concentrated fractions, enzymatic reactions were performed with β-casein as substrate. Assays were performed as described above and β-casein degradation and 11-mer formation were monitored by HPLC.

#### Optimum pH

The protease assay was performed at different pH values with purified protease. A β-casein solution (2% w/v) was incubated with purified protease at 50°C (optimum temperature) for 72 h. The hydrolysis reaction was carried out in a solution with purified protease (10 mL), β-casein solution (2% w/v; 20 mL) and PBS buffer (70 mL), at pH 4, 6, 7, 8, and 10. After incubation, samples were boiled for 10 min to stop the reaction, filtered through a Millipore 0.45 μm pore-size filter and 11-mer peptide was analyzed by HPLC.

#### Optimum Temperature

The protease assay was performed with purified protease at different temperatures. The β-casein degradation assay was carried out as above, during 72 h, at pH 6.4 and at temperatures of 35, 40, 50, and 60°C. As in the case of pH studies, the amount of 11-mer peptide was analyzed by HPLC.

#### Influence of Zinc, Potassium, and Calcium

The influence of zinc, potassium, and calcium on the purified protease activity was studied. As above, the assay was carried out with β-casein (2% w/v) as substrate and 70 μL citrate-phosphate buffer (pH 6.4, 50 mM). Incubation conditions were 50°C (optimum temperature) for 72 h. Minerals were added separately at a final concentration of 10 mM. After incubation, samples were boiled and 0.45 μm pore-size filtered, and 11-mer was analyzed by HPLC. A control was included without the addition of any mineral.

### Statistical Analysis

In all cases, results obtained were analyzed using GraphPad Prism 4 software (GraphPad software, La Jolla, CA, USA). Data were subjected to one-way analysis of variance (ANOVA). The Tukey’s multiple comparison test was used for comparison of means.

## Results

### Purification and Identification of the Substance of Interest by Cationic Exchange Followed by Reverse-Phase Chromatography

#### Identification of the Nature of the Active Compound

In order to determine the nature of the active compound responsible for inhibition of rotavirus, the freeze-dried samples were treated with proteinase K, pepsin and lipases. Non-treated CECT 7210 supernatant significantly decreased infection via either strategy A or B, by 51.6 and 83%, respectively (**Table 2**). Analysis of rotavirus SA11 proliferation for the aliquots processed by lipases showed no changes in supernatant rotavirus inhibition values (data not shown). Supernatant treated with proteases showed no inhibition of rotavirus infection via either strategy A or B (**Table 2**) and confirmed the proteinaceous nature of the active compound.

**Table 2:** Assay for *in vitro* activity against human rotavirus SA11 in MA-104 cell line in supernatants obtained with the growth of *B. longum* subsp. *infantis* CECT 7210, and treated.

Assays	% Focus of infection ± SD	
	Strategy A	Strategy B
Pepsin treated supernatant of CECT7210	134.6 ± 26.7	117.9 ± 26.8
Proteinase K treated supernatant of CECT7210	117.9 ± 33.4	101.8 ± 7.6
Untreated CECT 7210 supernatant	48.4 ± 30.7^a^	17.0 ± 4.1^b^
Assay positive control	100.0 ± 32.2	100.0 ± 33.3
Negative control QC3	121.7 ± 7.14	96.4 ± 16.3
Negative control C1	100.0 ± 16.1	100.0 ± 33.3

*Results are the average of three different experiments and are expressed as percentage of infectious foci. SD, standard deviation; ^a^*P*-value < 0.05; ^b^*P*-value < 0.01.*

#### Purification and Identification of the Active Substance against Rotavirus

**FIGURE 1:**
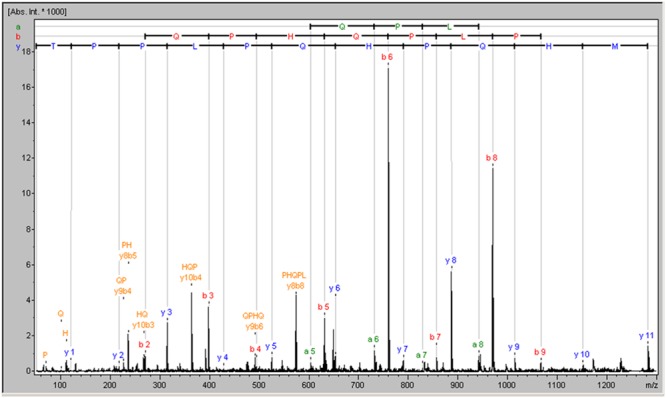
**MALDI mass spectrum of the common peptide among functional fractions obtained following purification by cationic exchange chromatography**.

The strategy used to identify the active substance against rotavirus started with its purification from the CECT 7210 active supernatant. To do this, a total of 28 fractions of reverse phase were selected for rotavirus inhibition assays (strategy B). Of these, only six fractions rendered a significant reduction in infected foci and were selected for further investigation. The selected fractions are summarized in **Table 3**. All the fractions that proved positive for inhibition were analyzed by MALDI-TOF peptide mass fingerprinting to determine the molecular weights of the peptide/s present in each fraction (results of each fraction are not shown). Analyzing all the spectra, the only common signal in all the selected fractions corresponded to a peptide with a molecular weight of 1,282.63 Da. To identify the molecule with potential activity against rotavirus, the signal was identified by *de novo* sequencing, and the result was a peptide of 11-amino acids whose sequence was MHQPHQPLPPT (named 11-mer peptide) and whose molecular mass was 1282.63 Da (**Figure 1**). This sequence was checked against the data base of proteins/peptides by means of the BLAST online tool^1^, and was identified as a part of the cow milk β-casein, corresponding to amino acids sequence (position 158–169; *Bos taurus* β-casein, GenBank accession no. AAA30431.1, gi: 162805).

**Table 3:** Inhibition of rotavirus SA11 infection in MA-104 cell line by selected reverse phase fractions following strategy B.

Sample	Fraction of cationic exchange	% Focus of infection ± SD
2/13	2	54.4 ± 12.1^a^
3/9	3	56.9 ± 29.3^a^
3/10		23.6 ± 16.6^b^
4/8	4	25.8 ± 8.9^b^
4/11		27.9 ± 8.6^b^
4/27		70.5 ± 18.7
Control		100.0 ± 10.9

*Results are the average of at least two independent experiments. SD, standard deviation; ^a^*P*-value < 0.05; ^b^*P*-value < 0.001.*

#### Analysis of the Inhibition of Human Rotavirus Proliferation by 11-mer Peptide

**FIGURE 2:**
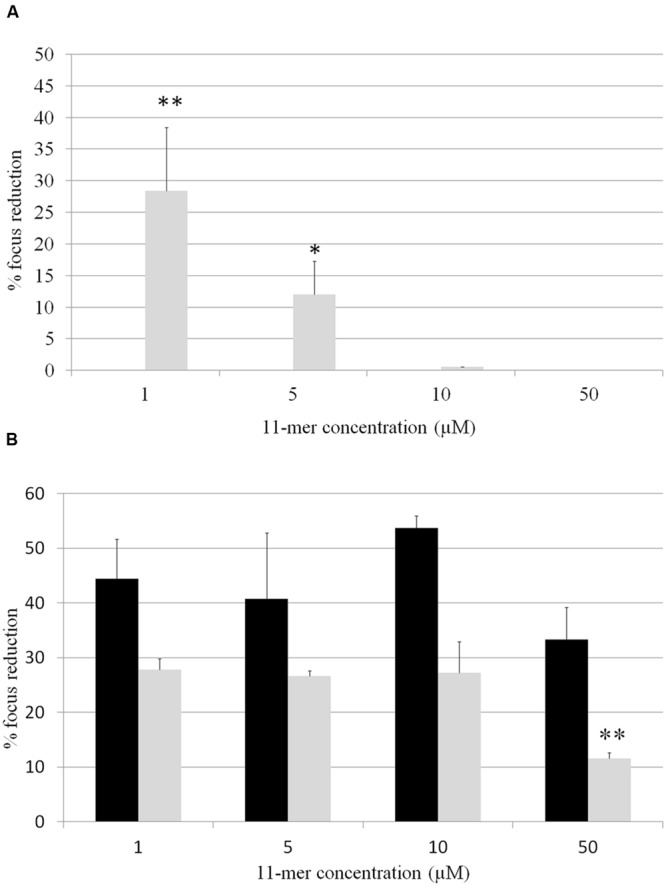
**Percentage of focus reduction in human rotavirus infection by strategy A, in which 11-mer peptide was incubated with rotavirus followed by the infection of the cells with this virus-compound mixture.** Black bars correspond to MA-104 cell line and gray bars to HT-29 cell line. **(A)** Wa virus assays; **(B)** Ito virus assays. No inhibition was obtained for virus Va70. Data are the average of at least two independent experiments. Error bars represent the standard errors for replicates. ^∗^*P*-value < 0.05; ^∗∗^*P*-value < 0.01.

**FIGURE 3:**
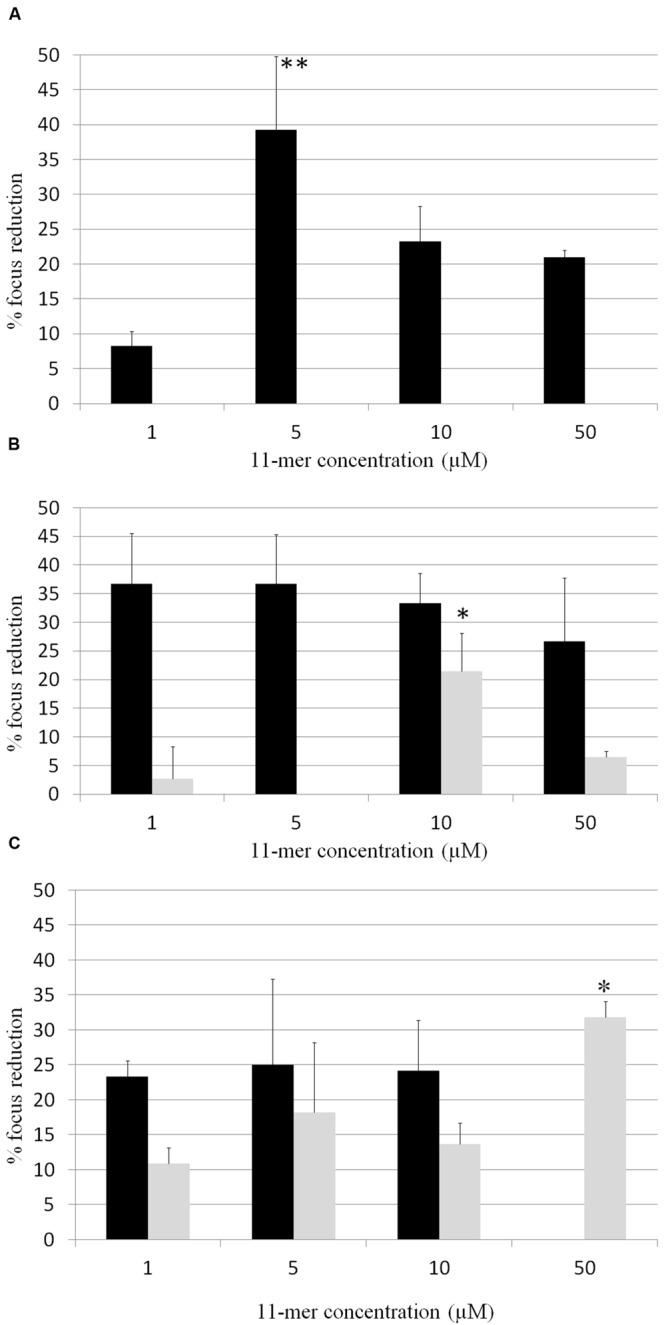
**Percentage of focus reduction in human rotavirus infection by strategy B, in which 11-mer peptide was incubated with the cell cultures followed by the inoculation of the virus.** Black bars correspond to MA-104 cell line and gray bars to HT-29 cell line. **(A)** Wa virus assays; **(B)** Ito virus assays; **(C)** VA70 virus assays. Data are the average of at least two independent experiments. Error bars represent the standard errors for replicates. ^∗^*P*-value < 0.01; ^∗∗^*P*-value < 0.001.

In order to determine whether 11-mer peptide was responsible for rotavirus inhibition, assays with human rotavirus Wa, Ito, and VA70 were performed in MA-104 and HT-29 cell lines with the synthesized peptide, and following both strategy A (tested compounds were incubated with rotavirus prior to cells infection; **Figure 2**) and B (cells were incubated with tested compounds prior to cells infection; **Figure 3**). Results were different in the case of each virus and cell line. In general, inhibition percentages were higher in assays performed in MA-104 cell line. Taking into account peptide concentration, inhibition results reached a plateau at lower concentrations (1 μM) and did not improve with higher peptide amounts. This result may be due to the potential formation of 11-mer peptide aggregates at higher concentrations, which leads to a loss of functionality depending on virus and cell line. For strategy A, no inhibition was obtained in VA70 assays. In the case of Wa virus assays, the highest inhibition percentage was 28.4% foci reduction at 1 μM 11-mer concentration (*p*-value < 0.001). In the case of Ito virus, foci reduction ranged from 33.3 and 53.7% in MA-104 cell line and from 11.6 and 26.8% in HT-29 cells. Following strategy B, Wa, Ito, and VA70 virus infections were inhibited for MA-104 cell line assays, the highest result being a 39.3% foci reduction at 5 μM 11-mer concentration (Wa virus). In HT-29 cells, no inhibition was obtained with Wa virus, and inhibition rendered its maximum for VA70 virus at 50 μM 11-mer concentration (31.8% foci reduction, *p*-value < 0.01).

### Purification and Identification of the Protease

As human rotavirus proliferation is thought to be inhibited by the 11-mer peptide (found in cow milk β-casein), we evaluated a hypothetical mechanism whereby the peptide is released by a potential supernatant protease activity, which hydrolyses the casein present in MRS broth. To check this hypothesis, a strategy was adopted to purify and identify the potential enzyme. First of all, supernatant was chromatographically purified and the final protease-positive fraction was electrophoresed. The single 47 KDa band present in the SDS-PAGE gel was separated and analyzed by MALDI-TOF peptide mass fingerprinting. The band was identified as “MalE-type ABC sugar transport system periplasmic component,” being the highest homology with sequence gi| 189440352 (MalE-type ABC sugar transport system periplasmic component from *B. longum* DJO10A), which has a molecular weight of 47.155 KDa. In order to evaluate the formation of 11-mer peptide from β-casein by the activity of the purified protease, a hydrolysis assay was performed using the purified protease as enzyme and β-casein as substrate. After 48 h of hydrolysis, peptide quantification by HPLC showed an efficiency of 28 ± 1 μg of 11-mer peptide/mL.

### Characterization of the Purified Protease

The purified protease was characterized in terms of carbon source, pH, temperature, and cofactors. Protease activity from *B. longum* CECT 7210 supernatants of media containing glucose, lactose, and maltose as sole carbon source is shown in **Table 4**. In all cases, significant higher activity was found in supernatants in which the probiotic strain *B. longum* CECT 7210 was grown in the presence of maltose. The supernatants rendered the highest value at 3 h of incubation in media with maltose, with an activity of 36.11 mU/mL (*p*-value < 0.01). Regarding the purified fraction, activity values were around 28 times higher, with a maximum of 1005.83 mU/mL of activity, at 2 h of incubation (*p*-value < 0.001). **Table 4** summarizes peptide quantification obtained when supernatants and concentrated fractions were incubated with β-casein as substrate. Results ranged from 39 to 110 μg of 11-mer peptide/mL, with the highest value corresponding to supernatants and fractions obtained from the growth of probiotic CECT 7210 with maltose as sole carbon source (90 and 110 μg peptide/mL, respectively).

**Table 4:** Protease activity and 11-mer production of both supernatants and concentrated fractions (higher than 3 KDa) in supernatants obtained with the growth of *B. longum* subsp. *infantis* CECT 7210 in the presence of different carbohydrates, at different times of incubation.

		Protease activity ± SD (mUI/mL)			11-mer production ± SD (μg/mL)		
	Time of incubation	Glucose	Lactose	Maltose	Glucose	Lactose	Maltose
Supernatant	1 h	1.67 ± 1.41	2.67 ± 1.89^a^	15.33 ± 0.94^a^	n.t.	n.t.	n.t.
	3 h	1.11 ± 1.57	12.22 ± 3.14^b^	36.11 ± 2.36^a^	n.t.	n.t.	n.t.
	5 h	5.33 ± 0.94	9.00 ± 0.47^b^	30.00 ± 0.94^a^	47 ± 4	65 ± 10	90 ± 6^b^
>3 KDa fraction	1 h	81.67 ± 11.36	283.33 ± 47.14^b^	973.33 ± 202.70^a^	n.t.	n.t.	n.t.
	2 h	153.33 ± 16.50	508.33 ± 9.43^a^	1005.83 ± 48.31^c^	39 ± 7	73 ± 13	110 ± 45

*Results are the average of at least two different experiments. SD, standard deviation; n.t., not tested; ^a^*P*-value < 0.01; ^b^*P*-value < 0.05; ^c^*P*-value < 0.001.*

Results regarding the effect of pH and temperature in 11-mer production from the purified protease are summarized in **Table 5**. The maximum 11-mer peptide concentration was 21 μg/mL. This value was obtained at pH 7–8. No significant differences were obtained at pH 4–10. Regarding the optimum reaction temperature, values obtained ranged from 20 to 22 μg of 11-mer peptide/mL, but no significant differences were found. Maximum concentration of 11-mer peptide was obtained at 50°C and no activity was found at 60°C.

**Table 5:** Effect of pH and temperature in 11-mer production by using protease-extract as enzyme and β-casein as substrate.

11-mer (μg/mL) ± SD			
**pH**		**Temperature**	
pH 4	19 ± 3	30°C	21 ± 3
pH 6	20 ± 4	35°C	20 ± 4
pH 7	21 ± 3	40°C	22 ± 5
pH 8	21 ± 4	50°C	21 ± 2
pH 10	20 ± 3	60°C	n.d.

*Data are the average of at least two independent experiments. n.d., not detected.*

In order to characterize the purified protease thoroughly, we analyzed the influence of zinc, potassium, and calcium as cofactors on 11-mer formation. Results are shown in **Table 6**, and ranged from 11 to 21 μg 11-mer peptide/mL, with the highest value corresponding to the assay without mineral addition.

**Table 6:** Evaluation of the effects of zinc, calcium, and potassium addition on the production of 11-mer peptide from β-casein.

Ions	% 11-mer peptide production ± SD
Control	100
Zn	57.65 ± 1.88^b^
Ca	82.54 ± 4.53^a^
K	82.63 ± 6.03^a^

*Control has no mineral addition. Data are the average of at least two independent experiments. SD, standard deviation; ^a^*P*-value < 0.01; ^b^*P*-value < 0.001.*

## Discussion

Recent clinical trials have demonstrated that some probiotic strains are able to ameliorate acute rotaviral diarrhea (Grandy et al., 2010; Lee et al., 2014). It is assumed that gut microbiota balance, the enhancement of mucosal barrier and the modulation of the immune response afford protection against rotavirus diarrhea (Kotzampassi and Giamarellos-Bourboulis, 2012). However, few studies have investigated the mechanisms underlying the protective effect of probiotics against rotavirus. A previous work demonstrated the direct effect of strain *B. longum* subsp. *infantis* CECT 7210 against rotavirus replication (Muñoz et al., 2011). Likewise, Lee et al. (2014) found that *B. longum* (IBG) and *L. acidophilus* (LA) strongly inhibited rotavirus infection using Vero cell line, but did not identify the mechanism further. In another study, Maragkoudakis et al. (2010) investigated the induction of NO- and H_2_O_2_ reactive oxygen species (ROS) released by cell lines co-incubated with lactic acid bacteria, and results were strain and cell line specific. Other data report that probiotics can block viral attachment by competitive inhibition if they are able to bind viral receptors at the surface of intestinal cells (Colbère-Garapin et al., 2007). In our study, a systematic approach was taken to identify the molecules directly involved in rotavirus inhibition.

First of all, to determine the nature of the substance of interest, supernatants with and without enzymatic treatment were analyzed for functionality. In these assays, the high percentage of inhibition of the non-treated cell-free supernatants suggested that part of the activity of CECT 7210 strain against rotavirus was not directly exerted by cells, and that the molecule responsible for direct rotavirus inhibition was released to the supernatant, as previously hypothesized for other probiotic strains (Botić et al., 2007). In assays performed to identify the nature of the functional compounds, treatment with different proteases eliminated inhibition capacity, confirming the proteinaceous nature of the substance responsible for inhibiting rotavirus infection. Similar assays were applied with lipases but inhibition capacity was not reduced (data not shown), refuting the potential lipidic nature of the substance of interest.

The direct effect of different compounds such as polyphenols (Kwon et al., 2010) or theaflavins (Clark et al., 1998) against rotavirus have been reported previously; however, the role played by molecules of a proteinaceous nature against rotavirus has been poorly described. In the literature, the most frequently reported protein is lactoferrin, which confers at least part of the antiviral properties of breast milk (van der Strate et al., 2001), and prevents the adsorption of rotavirus to the target cell due to its capacity to bind virus particles (Superti et al., 1997).

Once we had confirmed the proteinaceous nature of the substance acting against rotavirus, we went on to identify the active compound. We performed a supernatant purification strategy based on cationic exchange chromatography. This strategy had been carried out successfully to purify a probiotic antibacterial compound (Chenoll et al., 2011). Following this strategy, 28 fractions with cationic nature were obtained. Based on the inhibition of rotavirus infection, only six fractions were positive and thus selected for MALDI-TOF further analysis. Their spectra pointed to one functional compound, the 11-amino acid peptide MHQPHQPLPPT (11-mer), common in all the positive fractions. This peptide is part of cow milk β-casein and has a molecular mass of 1.28263 KDa. In order to verify the functionality of the 11-mer peptide, inhibition assays were run with the synthesized peptide in Wa, Ito, and VA70 rotavirus using both HT-29 and MA-104 cell lines. The results were similar to those previously published with the probiotic *B. longum* CECT 7210 (Muñoz et al., 2011), reaching a maximum of 56% of infectious foci inhibition, and clearly confirming peptide 11-mer as being responsible for rotavirus inhibition. Reports on the direct activity of peptides against rotavirus are very scarce. Ijaz et al. (1998) found that a synthetic peptide from VP4 directly inhibited rotavirus. Their study hypothesized that VP4-peptide blocks the receptor sites on the host cells. In other studies, Kvistgaard et al. (2004) and Bojsen et al. (2007) found that some bovine macromolecular whey proteins may exert *in vitro* and *in vivo* inhibitory activity against rotavirus, the major component being bovine IgG. Regarding lactoferrin, few peptides have been reported. Superti et al. (1997) used different peptides coming from artificially hydrolyzed lactoferrin to demonstrate that some peptides are able to interfere with different stages in virus replication, and the importance of specific recognition events. Regarding casein as the source of antiviral activity, previous work in bovine milk demonstrated the antiviral activity of κ-casein against human rotavirus and concluded that the inhibitory mechanism of bovine κ-casein against human rotavirus involves direct binding to viral particles via glycan residues (Inagaki et al., 2014). In our case, the 11-mer peptide does not contain glycan residues, and furthermore, its antiviral activity is conserved with synthesized peptide (with no glycan residues). Therefore, another mechanism must be involved in its activity against rotavirus. The facts that the peptide was effective in assays carried out with both strategies (A and B), and that we found different sensitivities depending on the virus and cell line, strongly suggest a two-way effect, in which the interaction of the peptide with both the mammalian cell surface and directly with the virus, must play a role in the inhibition mechanism, as hypothesized previously (Muñoz et al., 2011). Moreover, positive results were obtained even at low peptide concentrations (1 μM), showing the peptide exerts a strong activity.

To identify the source of the 11-mer peptide, the analysis of all the experimental data lead us to hypothesize a secondary origin of the peptide, derived from the preliminary digestion of casein present in the commercial MRS growth medium used. A protease secreted by the probiotic strain *B. longum* subsp. *infantis* CECT 7210 may release 11-mer peptide by hydrolyzing the casein present in the culture. A mechanism by which functional molecules are released from casein was previously described by Janer et al. (2005) in *B. animalis* subsp. *lactis* species. These authors described a zinc metallopeptidase (PepO) able to hydrolyze α_s1_-casein (f1-23), which was suggested to play a role in the increased growth of *B. animalis* subsp. *lactis* in milk. Accordingly, we performed the purification and identification of the protease responsible for 11-mer production. To obtain the protease, we applied a strategy based on fractionation by anionic chromatography. Following this protocol, a protease-positive anionic extract was obtained, and a SDS-gel band confirmed a unique band. MALDI-TOF peptide mass fingerprinting analysis identified the purified protease as “MalE-type ABC sugar transport system periplasmic component.” This is a periplasmic binding protein, part of the Maltose ABC sugar transport system, which is involved in the transport of maltose and maltodextrins. Although preliminary studies with this component were performed in *Salmonella enterica* subsp. *enterica* serovar Typhimurium and *Escherichia coli* (reviewed in Ehrmann et al., 1998), it has been found in other bacteria, including bifidobacteria (Balac_0483 in assembly NC_012814.1 from *B. animalis* subsp. *lactis* Bl-04, BLD_1008 in assembly ACD98454.1 from *B. longum* DJO10A). With the data obtained by the whole genome sequencing of *B. longum* CECT 7210 strain (Chenoll et al., 2015; genome reference LN824140), the protein has been identified in its genome, with a molecular weight of 46.89 KDa, confirming that the protein is produced by this probiotic strain. Once identified, the MalE-type ABC sugar transport system periplasmic component was characterized in terms of its protease activity. The results obtained in inhibition studies with glucose, lactose, and maltose supernatants showed the highest activity when the probiotic was grown in maltose as the only carbon source. Moreover, maltose-grown purified fractions were around 28 times higher, with a maximum of 1000 mU mL^-1^ of activity. These results, together with the quantification of 11-mer peptide when these supernatants and fractions were incubated with β-casein, confirmed that protease activity is enhanced when maltose is the sole carbon source. This supports the identification of the MalE-type ABC sugar transport system periplasmic component as the molecule responsible for this protease activity. Although protease activity in MalE has not been described previously, our data point to a mechanism whereby the formation of the 11-mer peptide from β-casein is caused by the direct action of the purified protein, with an efficiency of 28 ± 1 μg/mL of reaction. Finally, optimum pH and temperature for purified protease activity have been established and the influence of zinc, potassium, and calcium as cofactors on 11-mer production discarded.

To our knowledge, this is the first report of a mechanism by which a probiotic produces a functional molecule exerting a direct activity against rotavirus. This mode of action supports the potential use of strain CECT 7210 in dairy infant formulas, in which functional peptide 11-mer would be produced by the probiotic from cow milk β-casein.

In summary, the results presented here are the first to identify a naturally produced peptide, the 11-mer (MHQPHQPLPPT), as the molecule directly responsible for rotavirus inhibition demonstrated by the probiotic *B. longum* subsp. *infantis* CECT 7210. The data reported here point to a mechanism based on the MalE-type ABC sugar transport system periplasmic component, which is able to exert a protease activity and to hydrolyze β-casein to produce the functional 11-mer peptide.

## Author Contributions

EC designed the experiments, participated in assays for hydrolyzing, fractioning, and identification of the supernatant molecules with protease and antirotaviral activity, and contributed to data-analysis and writing. BC participated in all the experiments. EB participated in hydrolyzing, supernatant fractioning, and identification of the protease experiments. JB designed and coordinated virus experiments and contributed to data-analysis and writing. DR designed and coordinated supernatant protease experiments, and contributed to data-analysis and writing. SG designed and coordinated protease and supernatants experiments, and contributed to data-analysis and writing. JF contributed to data-analysis and writing. MR participated in the design of experiments, and contributed to data-analysis and writing. JM designed the research plan and organized the study, and contributed to data-analysis and writing.

## Conflict of Interest Statement

This work was partially funded by Laboratorios Ordesa. JF, MR, and JM are employees of Laboratorios Ordesa. EC, BC, DR, and SG are employees of Biopolis.

## Footnotes

1 http://blast.ncbi.nlm.nih.gov/Blast.cgi
